# MMP7 cleaves remyelination‐impairing fibronectin aggregates and its expression is reduced in chronic multiple sclerosis lesions

**DOI:** 10.1002/glia.23328

**Published:** 2018-03-30

**Authors:** Peng Wang, Rianne P. Gorter, Jenny C. de Jonge, Muhammad Nazmuddin, Chao Zhao, Sandra Amor, Dick Hoekstra, Wia Baron

**Affiliations:** ^1^ Department of Cell Biology University of Groningen, University Medical Center Groningen Groningen the Netherlands; ^2^ Wellcome Trust – Medical Research Council Cambridge Stem Cell Institute and Department of Clinical Neurosciences University of Cambridge Cambridge United Kingdom; ^3^ Department of Pathology VU Medical Center Amsterdam the Netherlands; ^4^ Queen Mary University of London, Blizard Institute, Barts and The London School of Medicine and Dentistry London United Kingdom

**Keywords:** fibronectin aggregates, macrophage, microglia, MMP7, multiple sclerosis

## Abstract

Upon demyelination, transient expression of fibronectin precedes successful remyelination. However, in chronic demyelination observed in multiple sclerosis (MS), aggregates of fibronectin persist and contribute to remyelination failure. Accordingly, removing fibronectin (aggregates) would constitute an effective strategy for promoting remyelination. Matrix metalloproteinases (MMPs) are enzymes known to remodel extracellular matrix components, including fibronectin. Here, we examined the ability of MMPs to degrade fibronectin aggregates. Our findings reveal that MMP7 cleaved fibronectin aggregates resulting into a prominent 13 kDa EIIIA (16 kDa EDA)‐containing fragment. MMP7 was upregulated during lysolecithin‐induced demyelination, indicating its potential for endogenous fibronectin clearance. In contrast, the expression of proMMP7 was substantially decreased in chronic active and inactive MS lesions compared with control white matter and remyelinated MS lesions. Microglia and macrophages were major cellular sources of proMMP7 and IL‐4‐activated, but not IFNγ+LPS‐activated, microglia and macrophages secreted significant levels of proMMP7. Also, conditioned medium of IL‐4‐activated macrophages most efficiently cleaved fibronectin aggregates upon MMP‐activating conditions. Yet, coatings of MMP7‐cleaved fibronectin aggregate fragments inhibited oligodendrocyte maturation, indicating that further degradation and/or clearance by phagocytosis is essential. These findings suggest that MMP7 cleaves fibronectin aggregates, while reduced (pro)MMP7 levels in MS lesions contribute to their persistent presence. Therefore, upregulating MMP7 levels may be key to remove remyelination‐impairing fibronectin aggregates in MS lesions.

## 1 INTRODUCTION

Myelination of axons is not only essential for saltatory nerve conduction, but also required for neuronal survival as myelin provides trophic and metabolic support (Irvine & Blakemore, 2008; Fünfschilling et al., 2012). Hence, the persistent loss of intact myelin, as observed in the chronic demyelinating disease multiple sclerosis (MS), promotes secondary axonal degeneration, significantly contributing to disease progression (Compston & Coles, 2008; Irvine & Blakemore, 2008). Therefore, regeneration of myelin (remyelination), is essential to support and retain axons, and to prevent progressive decline.

Remyelination is dependent on precise and timely regulation of local signalling events, which control recruitment of activated oligodendrocyte progenitor cells (OPCs) to the demyelinated area and their subsequent differentiation into myelinating oligodendrocytes (Franklin & ffrench‐Constant, 2008). The extracellular matrix (ECM) is a major component of the signaling microenvironment and participates in the regulation of OPC behavior, its remodeling serving as an effective mechanism to regulate repair. Specifically, following central nervous system (CNS) demyelination, extensive ECM remodeling leads to alterations in ECM expression profiles (Back et al., 2005; Zhao, Fancy, Franklin, & ffrench‐Constant, 2009; Lau et al., 2012; Hibbits, Yoshino, Le, & Armstrong, 2012; Stoffels et al., 2013). For example, following lysolecithin‐induced demyelination and in chronic‐relapsing (cr‐)EAE, remyelination‐impairing fibronectin and chrondroitin sulfate proteoglycans (CSPGs), and remyelination‐promoting laminin are readily expressed (Milner et al., 2007; Lau et al., 2012; Stoffels et al., 2013). Evidently, the regulation and transient expression of these distinct ECM molecules, is essential in maintaining the proper physiological environment for timely development of OPCs into mature, myelinating oligodendrocytes. Indeed, a dysbalance in expression of these ECM entities contributes to an impaired differentiation of OPCs, as observed in 70% of MS lesions (Lucchinetti et al., 1999; Kuhlmann et al., 2008; Chang et al., 2012). More specifically, in chronic MS lesions, but not in toxin‐induced lesions, fibronectin is persistently present as aggregates, which frustrates OPC differentiation, and thereby impedes remyelination (Stoffels et al., 2013). Moreover, fibronectin precludes the ability of laminin, also present in MS lesions (Van Horssen, Bö, Vos, Virtanen, & de Vries, 2005), to facilitate myelin membrane formation (Baron et al., 2014), emphasizing the necessity to remove aggregated fibronectin to allow remyelination.

Interestingly, fibronectin mRNA is hardly present in chronic MS lesions (Stoffels et al., 2013) and aggregates are formed extracellularly (Mao & Schwarzbauer, 2005, our unpublished observations), indicating that a perturbed clearance, rather than an altered expression of fibronectin, is responsible for its accumulation. Selective removal of astrocyte‐derived fibronectin from the lesion site reveals that although dimeric fibronectin promotes OPC proliferation following demyelination, reduced numbers of OPCs suffice for successful remyelination (Stoffels, Hoekstra, Franklin, Baron, & Zhao, 2015). Therefore, removal of dimeric or aggregated fibronectin may represent a therapeutic strategy to promote remyelination in MS.

Important players in controlled ECM degradation are matrix metalloproteinases (MMPs). Commonly, MMPs are synthesized and secreted as pro‐enzymes that are subsequently activated by various proteinases, including other MMPs and plasmin (Lu, Takai, Weaver, & Werb, 2011). Under healthy conditions, MMPs are transiently involved in the regulation of ECM dynamics upon injury, and are under strict (local) control at various levels, including gene transcription, synthesis, secretion, pro‐peptide activation, and inhibition by physiological tissue inhibitors of MMPs (TIMPs). MMPs are implicated in the pathogenesis of MS, and some MMPs, including MMP3, MMP7, and MMP9, are upregulated in MS lesions (Cuzner et al., 1996; Maeda & Sobel, 1996; Cossins et al., 1997; Anthony et al., 1997; Lindberg et al., 2001). While fibronectin is a substrate for at least 14 distinct MMPs (Lu et al., 2011), it is unknown whether they dissociate, cleave and/or degrade fibronectin aggregates.

Given the role of MMPs in ECM remodeling in injured CNS, we examined here whether a perturbed expression and/or malfunctioning of MMP3, MMP7 or MMP9 contributes to the inability to clear dimeric fibronectin and/or fibronectin aggregates in MS lesions. We demonstrate that MMP7 cleaved fibronectin aggregates and that proMMP7 is weakly expressed in chronic MS lesions compared with remyelinated lesions. IL‐4‐activated microglia and macrophages were major cellular sources of proMMP7. Hence, local targeting of MMP7 levels in chronic MS lesions may represent a first step to remove remyelination‐inhibiting fibronectin aggregates.

## 2 MATERIALS AND METHODS

### 2.1 MS lesions

Autopsy samples of human brain material were obtained from the Netherlands Brain Bank and with the approval of the VU University Medical Ethical Committee (Amsterdam, The Netherlands). Patients and controls, or their next of kin, had given informed consent for the use of their brain tissue and clinical details for research purposes. For immunohistochemical analysis paraffin‐embedded (*n* = 12) or snap‐frozen (*n* = 5) tissue from MS patients and non‐neurological controls (*n* = 3) were used. Western blot studies were performed on nine control white matter (CWM), eight (chronic) active MS lesion [(c)aMS)], nine chronic inactive MS lesion (ciMS), and two remyelinated MS lesion (rMS) homogenates. Brain tissue was homogenized as previously described (Maier et al., 2007). CWM did not show any histological signs of inflammation and demyelination and was obtained from subjects without clinical signs of neurological disease. MS lesions were classified as previously described (van der Valk & De Groot, 2000).

### 2.2 Toxin‐induced demyelination

#### 2.2.1 Lysolecithin

To induce local demyelination 8–10‐week‐old female C57BL/6 mice (for RT‐qPCR) or Sprague Dawley rats (for Western blot) were injected with 1 µl of 1% lysolecithin (Sigma) in spinal cord white matter (Zhao, Li, & Franklin, 2006). At indicated time points, animals were sacrificed and tissue processed for Western blot and qPCR analysis as previously described (Zhao et al., 2006; Stoffels et al., 2013). For immunohistochemistry, animals were perfused with 4% paraformaldehyde via the left ventricle, after which the dissected spinal cord containing lesions was treated with 20% sucrose in phosphate‐buffered saline (PBS) overnight. The brains were cryosectioned at 12 μm thickness and stored at −80°C until further processing. Control spinal cord tissues were taken from non‐lesioned thoracic segments of spinal cord, distant from the lesion site. Experiments were performed in compliance with UK Home Office regulations.

#### 2.2.2 Cuprizone

To induce robust and reproducible demyelination of the *corpus callosum*, 9‐week old male C57BL/6 mice (Harlan, Horst, the Netherlands) were individually housed and subjected to a standard powder chow diet containing 0.2% cuprizone (bis(cyclohexanone)‐oxaldihydrazone, Sigma, St. Louis, MO). After 5 weeks, animals returned to standard chow. Tissue was processed as described for lysolecithin‐induced lesions. Demyelination of the *corpus callosum* was confirmed by Sudan black staining (0.1% in 70% ethanol for 5 min). All experimental procedures were approved by the Animal Ethical Committee of the University Medical Center Groningen (the Netherlands).

### 2.3 Primary cell cultures

#### 2.3.1 CNS‐resident cells

Primary cultures were derived from forebrains of 1–2 day‐old Wistar rats (Charles River) as described (Bsibsi, Nomden, van Noort, & Baron, 2012). A single cell suspension was obtained by mechanical and enzymatic (papain) digestion. Cells were cultured for 10–12 days on poly‐l‐lysine (PLL, 5 µg/ml, Sigma)‐coated tissue culture flasks (Nalge Nunc, Naperville, IL). OPCs and microglia grown on top of an astrocyte monolayer were isolated by a shake‐off procedure. First, *microglia* were detached by rotation on an orbital shaker (Innova 4000, New Brunswick Scientific) at 150 rpm for 1 hr. Upon centrifugation for 5 min at 150*g*, cells were resuspended in microglia medium (DMEM [Life Technologies, Paisley, UK]; 10% fibronectin‐free fetal bovine serum [FBS, Capricorn, Ebsodorfergrund, Germany see below]; 1% antibiotics [Life Technologies] and 1% glutamine [Life Technologies]). Microglia were plated at a density of 2.0 × 10^6^ cells per 10‐cm dish (Corning, Lowell, MA) and cultured with rat recombinant macrophage colony‐stimulating factor (M‐CSF, 10 ng/ml, Peprotech, London, UK). After 3 days, microglia were gently scraped, centrifuged for 5 min at 150*g*, and plated in 6‐well‐plates at a density of 1.0 × 10^6^ cells per well (in 1 ml). Microglia (>95% Iba1‐positive) were left untreated, or treated with either rat IFNγ (400 ng/ml, Peprotech) and lipopolysaccharide (LPS from *E.coli* O111:B4, 200 ng/ml, Sigma), or rat IL‐4 (40 ng/ml, Peprotech) for 6 hr (qPCR analysis) or 48 hr (Western blot analysis).

The mixed glia flasks were shaken overnight on the orbital shaker at 240 rpm, and the floating *OPCs* obtained by this procedure were further purified via differential adhesion (Bsibsi et al., 2012). Isolated OPCs (>97% Olig2‐positive) were plated at a density of 1.0 × 10^6^ per 10‐cm dish (in 6 ml) for Western blotting or on 8‐well Permanox chamber slides (Nunc) at a density of 30,000 cells per well for the maturation assays. OPCs were cultured for 2 days in SATO medium (Maier, Baron, & Hoekstra, 2005) supplemented with growth factors FGF‐2 (10 ng/ml, Peprotech) and PDGF‐AA (10 ng/ml, Peprotech). To obtain mature *oligodendrocytes*, OPC maturation was initiated by growth factor withdrawal and culturing in SATO supplemented with 0.5% FBS for 7 days.

To obtain *astrocytes*, the remaining astrocyte monolayer in the mixed glia flasks was shaken on the orbital shaker at 240 rpm overnight, followed by one passage to T162 flasks. At 90% confluency, the astrocytes were trypsinized and plated in 6‐well plates at a density of 0.5 × 10^6^ cells per well (in 1 ml) in DMEM and 10% fibronectin‐free FBS (see below). Astrocytes (>97% Aldh1L1‐positive) were untreated, or treated with a pro‐inflammatory cytokine cocktail containing rat TNFα (10 ng/ml, Peprotech), rat IFNγ (400 ng/ml) and rat IL1β (10 ng/ml, Peprotech) for 6 (RT‐qPCR analysis) or 48 hr (Western blot analysis).

#### 2.3.2 Bone marrow‐derived macrophages

Macrophages were derived from the bone marrow of 1–2 day‐old Wistar rats (Charles River). The femorae and tibiae bone marrow were flushed with BMDM medium (Roswell Park Memorial Institute [RPMI]‐1640 medium [Life Technologies]). Subsequently, all suspensions were centrifuged for 5 min at 150*g*, and cell pellets were resuspended in BMDM medium supplemented with 10% fibronectin‐free FBS, 1% sodium pyruvate (Life Technologies), and 1% antibiotics. Macrophages (>95% isolectin‐B4‐positive) were plated at a density of 2.0 × 10^6^ cells per 10‐cm dish and cultured with rat M‐CSF (10 ng/ml) for 5–7 days. For experimental analysis, macrophages were plated and treated as described for microglia.

### 2.4 Generation of fibronectin aggregates

Deoxycholate (DOC)‐insoluble aggregated fibronectin was prepared from primary rat astrocytes or MS lesions homogenates. Astrocytes were plated at a density of 1 × 10^6^ cells per 10‐cm dish, and after 1 hr treated with Toll‐like receptor 3 agonist poly(I:C) (50 μg/ml, GE Healthcare, Munich, Germany). After 48 hr, astrocytes were removed by water‐lysis for 2 hr at 37°C. The remaining astroglial matrices were scraped into ice‐cold DOC buffer (2% deoxycholate [Sigma] in 20 mM Tris‐HCl supplemented with complete Mini Protease inhibitor cocktail [Roche, Mannheim, Germany], pH 8.0). For extraction of fibronectin aggregates from MS lesions, tissue homogenates (100 μg) were incubated in ice‐cold DOC buffer for 30 min. To separate DOC‐insoluble fractions that contain fibronectin aggregates from the suspension, centrifugation was performed at 16,300*g* for 30 min. The fibronectin aggregate‐containing pellet was washed three times in PBS, followed by resuspension in PBS with a syringe and 25‐gauge needle. The quality of the fibronectin aggregates, that is, the lack of dimeric fibronectin and/or smaller products, and the extent of aggregation, was routinely checked by Western blot.

### 2.5 Fibronectin‐free serum

Fibronectin was depleted from serum with a gelatin sepharose 4B column (GE healthcare) according to manufacturer's instructions. The generated fibronectin‐free serum was filtered (0.2 μm) and stored in aliquots at −20°C. The absence of fibronectin in serum was confirmed by Western blot.

### 2.6 Fibronectin degradation assay

Plasma bovine fibronectin (5 μg, Sigma), human cellular fibronectin (5 μg, Sigma) and rat and human fibronectin aggregates (5 μg, see above) were respectively incubated with 100 ng recombinant human active MMP3 (Abcam, Cambridge, UK), MMP7 (Millipore, Darmstadt, Germany), and MMP9 (Millipore, San Diego, CA) in 50 µl MMP‐reaction buffer (50 mM Tris‐HCl, 0.15 M NaCl, 5 mM CaCl_2_, 0.05% Brij‐35, pH 7.5) at 37°C for 24 hr. For Western blot analysis, the reaction was terminated by adding non‐reducing SDS sample buffer. For coating purposes, the enzymatic activity of MMP7 was terminated by heating at 95°C for 10 min. Alternatively, fibronectin structural variants were incubated with PBS or cell conditioned media (35 µl) in the presence or absence of general MMP‐activator, 4‐aminophenylmercuric acetate (APMA, 2 mM, Sigma) in 50 μl MMP reaction buffer and incubated at 37°C for 72 hr.

### 2.7 Lactate dehydrogenase and MTT assay

OPCs were cultured in 24‐wells plates at a density of 50,000 cells per well on the indicated substrates. After 48 hr the medium [lactate dehydrogenase (LDH) assay] and cells [3‐ (4,5‐Dimethylthiazol‐2‐yl)‐2,5‐diphenyltetrazolium bromide; MTT assay] were analyzed as described (Stoffels et al., 2013). Briefly, the release of LDH into the medium was measured using a commercial LDH assay kit (Roche) according to manufacturer's instructions. The effect on cell viability was determined with an MTT assay, for which cells were incubated with MTT diluted in culture medium (0.5 mg/ml, Sigma) for 4 hr. MTT‐formazan crystals were collected in dimethyl sulphoxide and absorption measured at 560 nm.

### 2.8 Immunohistochemistry

#### 2.8.1 Toxin‐induced lesions

Sections were blocked and permeabilized with PBS containing 5% normal donkey serum and 0.1% Triton‐X‐100. Sections were incubated with a mixture of primary antibodies (Table 1) overnight at 4°C, followed by a 2‐hr incubation with appropriate Alexa‐conjugated secondary antibodies. Nuclei were visualized by Hoechst 33342 or DAPI (1 µg/ml, Sigma). Sections were analyzed with confocal laser scanning microscopy (Leica SP8 AOBS).

**Table 1:** Primary antibodies used during WB, ICC, and IHC

	Company	WB	ICC/IHC	Antigen retrieval (ICC/IHC)	Blocking (ICC/IHC)
Anti‐actin (mAb)	Sigma	1:1000	n.a.	n.a.	n.a.
Anti‐arginase‐1 (mAb)	BD Bioscience	1:250	n.a.	n.a.	n.a.
Anti‐CD40 (mAb)	AbDSerotec	n.a.	1:800	no	no
Anti‐CD206 (mAb)	BD Bioscience	n.a.	1:800	no	no
Anti‐EIIIA‐fibronectin (mAb)	Sigma	1:500	n.a.	n.a.	n.a.
Anti‐fibronectin (pAb)	Millipore	1:1000	n.a.	n.a.	n.a.
Anti‐HLA‐DR (mAb)	Ebioscience	n.a.	1:1000 (single) 1:2000 (double)	Citrate buffer (pH 6.0)	no
Anti‐MBP (mAb)	Serotec	n.a.	1:250	no	4% BSA
Anti‐MMP3 (pAb)	Abcam	1:1000	n.a.	Citrate buffer (pH 6.0)	no
Anti‐MMP7 (pAb)	Gene Tex and Bioworld	1:1000	1:500	TRIS/EDTA (pH 9.0)	antibody diluent with 10% normal human serum
Anti‐MMP9 (pAb)	Torrey Pines Biolabs	1:1000	n.a.	n.a.	n.a.
Anti‐iNOS (mAb))	BD Biosciences	1:500	n.a.	n.a.	n.a.
Anti‐PLP (mAb)	Bio‐rad	n.a.	1:3000	no	no
Anti‐vimentin (mAb)	In house (V9)	n.a.	1:6000	Citrate buffer (pH 6.0)	no

n.a. = not applicable; mAb = monoclonal antibody; pAb = polyclonal antibody; WB = Western blot; ICC = immunocytochemistry; IHC = immunohistochemistry.

#### 2.8.2 MS lesions

Luxol‐fast blue staining was used to identify shadow plaques. To visualize myelin loss, microgliosis and (pro)MMP3 and (pro)MMP7 expression, 5‐µm serial sections of active (*n* = 3), chronic active (*n* = 3) and inactive lesions (*n* = 3), shadow plaques (*n* = 3) and healthy controls (*n* = 3) were single‐stained or double‐stained. Slides were deparaffinized in xylene and descending ethanol concentrations. After endogenous peroxidase inhibition with H_2_O_2_, antigen retrieval and/or blocking (Table 1), sections were incubated with primary antibodies for 1 hr or overnight (Table 1). The sections were washed and incubated with appropriate horseradish peroxidase‐conjugated secondary antibodies (Dako) for 1 hr and visualized with 3,3'‐diaminobenzidine (DAB, 1:50, Dako, Heverlee, Belgium) for 10 min. For single staining, sections were counterstained with haematoxylin, dehydrated in ascending concentrations of alcohol and xylene and mounted with Quick‐D mounting medium (Merck, Darmstadt, Germany). For double‐staining, slides were heated to detach the first primary antibody or directly incubated with appropriate alkaline phosphatase (AP)‐conjugated second primary antibody for 1 hr or overnight. AP‐labeled cells were visualized with liquid permanent red (Dako, 1:100) for 10 min. Sections were counterstained with haematoxylin and mounted with Aquatex (Merck). For MMP‐7 and CD40/CD206 double‐staining, 5 µm cryosections containing active MS lesions (*n* = 5) were dried overnight, fixated for 10 min in 4% paraformaldehyde and incubated with 0.1% glycine for 10 min. After blocking with H_2_0_2_, slides were incubated overnight with both primary antibodies (Table 1), which were visualized with DAB and liquid permanent red (LPR) as described above. Sections were analyzed and representative pictures were taken with an Olympus BX41 microscope, equipped with a Leica MC170 HD camera.

### 2.9 Immunocytochemistry

Cells were fixed with 4% paraformaldehyde in PBS for 20 min, permeabilized for 5 min in ice‐cold methanol and blocked with 4% BSA for 30 min. The cells were incubated for 1–2 hr with anti‐MBP, followed by incubation with an appropriate TRITC‐conjugated secondary antibody (1:50, Jackson ImmunoResearch, Westgrove, UK) and DAPI for 25 min. Cells were covered with mounting medium (Dako) to prevent image fading and analyzed with conventional immunofluorescence microscopy (Olympus AX70 or Leica DMI 6000 B). Oligodendrocytes were characterized by morphology, that is, cells with typical astrocytic morphology were excluded (<3%), and in each experiment at least 250 cells were manually scored as either MBP‐negative or MBP‐positive, while in addition MBP‐positive cells were classified as myelin membrane‐forming or non‐myelin membrane‐forming.

### 2.10 Western blot analysis

Cells were collected by scraping with PBS and centrifuged at 9,200*g* for 5 min. Cell pellets were sonicated in TNE buffer (50 mM Tris‐HCl, 150 mM NaCl, and 5 mM EDTA, pH 7.5) for 10 s on ice. Total protein concentration was measured by a Bio‐Rad DC Protein Assay (Bio‐Rad Laboratories, Herculas, CA) using BSA as standard. Equal amounts of protein (50 μg for cell lysates and brain homogenates) or equal volumes of medium (40 µl) were mixed with SDS‐reducing loading buffer, denatured at 95°C for 5 min and subjected to Western blotting as previously described (Bsibsi et al., 2012). Primary antibodies used are indicated in Table 1. The signals were detected using the Odyssey Infrared Imaging System (LI‐COR Biosciences, Lincoln, NE) and analyzed using Scion image software.

### 2.11 Real‐time quantitative polymerase chain reaction reaction (RT‐qPCR)

Total RNA was extracted from cells with the RNeasy Micro Kit (Qiagen, Hamburg, Germany), according to manufacturer's instructions. Total RNA (1 μg) was reverse transcribed using oligo (dT)_12–18_ (500 μg/ml, Invitrogen, Paisley, UK), 10 mM dNTP Mix (Invitrogen), 0.1 M dithiothreitol (DTT, Sigma), 5× first strand buffer and Moloney Murine Leukemia Virus Reverse Transcriptase (M‐MLV RT, Invitrogen). RT‐qPCR was performed by an Applied Biosystems 7900HT Real‐Time PCR System. For each reaction, 10 ng cDNA, 10 pM primers (Table 2) and absolute SYBR Green Rox Mix (ThermoScientific, Breda, the Netherlands) were mixed. Gene expression was calculated by the 2^‐ΔΔct^ method. GADPH and HMBS were used as housekeeping genes.

**Table 2:** Primer pair set sequences used during RT‐qPCR

	Forward primer	Reverse primer	Length (bp)
MMP2 (Rn)	GCTTCTGTCCTGACCAAG	CAGGGTCCTGAGAGTGTTC	90
MMP3 (Mm)	TGGAGATGCTACTTTGACG	AGCCTTGGCTGAGTGGTAGA	121
MMP3 (Rn)	GCGGGGAGAAGTCTTGTTCT	AGACGGCCAAAATGAAGAGA	100
MMP7 (Mm)	TAGGCGGAGATGCTACTTT	GTGGCAGCAAACAGGAAGTT	82
MMP7 (Rn)	CGGAGATGCTCACTTTGACA	CATGAGTGGCAACAAACAGG	83
MMP8 (Rn)	TTGGACATTCCTTGGGACTC	AGGTTGGACAGGGTTGTCTG	155
MMP9 (Rn)	TGTATGGTCGTGGCTCTAAAC	GTGGGACACATAGTGGGAG	91
MMP11 (Rn)	GGCAACTTGTAAGGGAGCAG	AAGTTGTCCCCATGCCAGTA	148
MMP12 (Rn)	TGGTACCTTAGCCCATGCTT	AGGAACAGGTTTGTGCCTTG	108
MMP13 (Rn)	GCCAGAACTTCCCAACCA	CCGCAGCACTGAGCCTT	176
MMP14 (Rn)	AATAAGTACTACCGCTTCATG	GAGACTCAGGGATTCCTTC	91
ADAMTS4 (Rn)	GCCCGATTCATCACTGACTT	GCGGTCAGCATCATAGTCCT	117
TIMP1 (Mm)	TCCCCAGAAATCAACGAGAC	CATTTCCCACAGCCTTGAAT	88
TIMP1 (Rn)	GGTTCCCTGGCATAATCTGA	ATGGCTGAACAGGGAAACAC	99
TIMP2 (Rn)	TGGACGTTGGAGGAAAGAAG	TCCCAGGGCACAATAAAGTC	97
TIMP3 (Rn)	GACCACAGCAGCTACCATGA	GCTTCTTTCCCACCACTTTG	167
TIMP4 (Rn)	TGCCAAATCACCACTTGCTA	ATAGAGCTTCCGTTCCAGCA	93
tPA (Rn)	ACGTACAGCTCCCTGACTGG	CAGCCGGTCAGAGAAGAAAG	86
uPA (Rn)	TGCCCAAAGAAATTCAAAGG	CGGCCTTTGGTGTCAGTATT	110
HBMS (Mm/Rn)	CCGAGCCAAGCACCAGGAT	CTCCTTCCAGGTGCCTCAGA	107
GAPDH (Mm/Rn)	CATCAAGAAGGTGGTGAAGC	ACCACCCTGTTGCTGTAG	204

Mm = mus muculus; Rn = rattus norvegicus; RT‐qPCR = real‐time quantitative analysis.

### 2.12 Statistics

Data are expressed as mean ± *SEM* for at least three independent experiments. Statistical analysis was performed with a one sample *t* test when relative values of conditions were calculated by setting the control as 1 in each independent experiment. When absolute values of two means were compared, statistical significance was calculated by Student's *t* test, and when more than two means were compared, by one‐way analysis of variance (ANOVA), followed by Newman‐Keuls multiple comparison test post‐test. In all cases, *p* < .05 was considered significant.

## 3 RESULTS

### 3.1 proMMP3 and MMP7 are upregulated upon toxin‐induced demyelination

**Figure 1:**
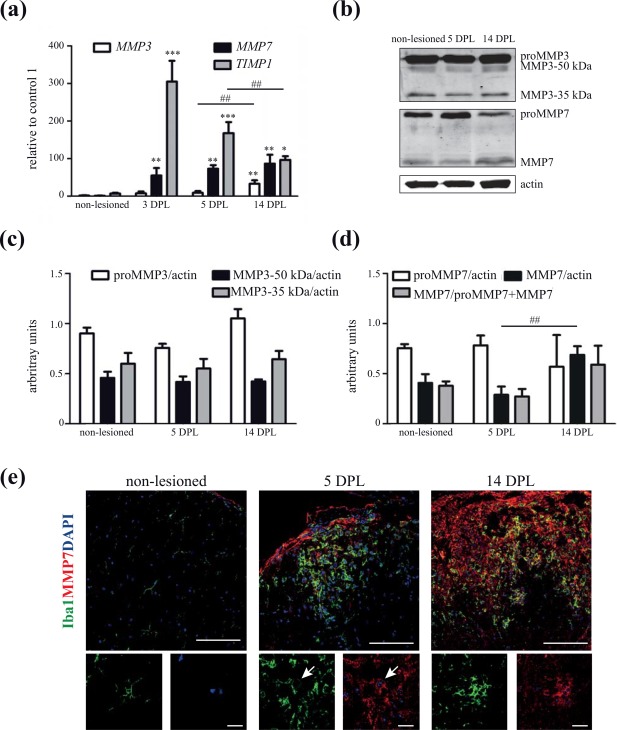
MMP expression upon lysolecithin‐induced demyelination. (a) Real‐time quantitative PCR analysis of (pro)MMP3, (pro)MMP7 and TIMP1 against GAPDH (shown) and HMBS (not shown, but yielding comparable findings) in non‐lesioned spinal cord white matter and lysolecithin‐induced lesions. DPL is days post lesion. Bars depict relative mean expression levels + *SEM* versus 1 control animal which was set at 1. Statistical differences as assessed with a one‐way ANOVA (compared with ctrl **p < .01, ***p < .001; compared with 14 DPL ##*p* < .01, Newman‐Keuls, *n* ≥ 3). (b) Western blot showing (pro)MMP3 and (pro)MMP7 expression in non‐lesioned and lysolecithin‐induced lesions (50 µg). Actin serves as a loading control. Representative blots of three animals per condition are shown. (c and d) Quantitative analysis of proMMP3 and 35 and 50 kDa MMP3 (c) and proMMP7, MMP7, and MMP7/proMMP7 + MMP7 (d) expression of b. Bars depict mean + *SEM* of the relative MMP expression to actin. Statistical differences as assessed with a one‐way ANOVA are indicated (*p < .05, Newman‐Keuls, *n* = 3). (e) Double staining of non‐lesioned and lysolecithin‐induced lesions for MMP7 (red) and the microglia marker Iba1 (green). Hoechst‐stained nuclei are indicated in blue. Scale bars are 100 or 20 μm (insets) [Color figure can be viewed at http://wileyonlinelibrary.com]

**Figure 2:**
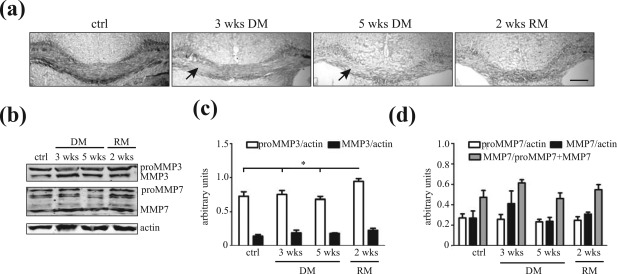
MMP expression upon cuprizone‐induced demyelination. (a) Sudan Black staining of control mice (ctrl), mice fed with 0.2% cuprizone for 3 or 5 weeks (3 or 5 wks DM), and mice 2 weeks after removal of 5 weeks cuprizone feeding (2 wks RM). Note the absence of Sudan Black staining, indicating prominent demyelination (DM), at the *corpus callosum* at 3 and 5 weeks, and the reappearance of Sudan Black staining, that is, remyelination (RM), upon 2 weeks of cuprizone removal. Scale bar is 200 µm. (b) Western blot showing (pro)MMP3 and (pro)MMP7 expression in total *corpus callosum* homogenates (50 µg) of mice indicated in a. Actin serves as a loading control. Representative blots of four animals per condition are shown. (c and d) Quantitative analysis of proMMP3 and MMP3 (c) and proMMP7, MMP7, and MMP7/proMMP7 + MMP7 (d) expression of b. Bars depict mean + *SEM*. Statistical differences as assessed with a one‐way ANOVA (*p < .05, Newman‐Keuls)

Immunohistochemical analysis and *in situ* hybridization studies revealed that expression of fibronectin‐degrading proteinases MMP3 and MMP7 is enhanced in active demyelinating MS lesions (Anthony et al., 1997; Cossins et al., 1997; Lindberg et al., 2001). To examine whether the enhanced expression of these MMPs in MS lesions is a natural response to a demyelinating insult, we first examined their expression levels in two different animal models of toxin‐induced demyelination. Notably, we and others have previously shown that upon toxin‐induced demyelination, dimeric fibronectin is transiently increased during demyelination, and its clearance commensurate with remyelination (Zhao et al., 2009; Hibbits et al., 2012; Stoffels et al., 2013; Espitia Pinzon et al., 2017), making it an ideal model to examine the regulation of “natural” fibronectin‐degrading proteolytic enzymes involved in ECM remodeling upon CNS demyelination. Focal demyelination in spinal cord white matter is induced by a single injection of the detergent lysolecithin, being maximal at 5 days post lesion (DPL), which is followed by robust remyelination at 14–21 DPL (Zhao et al., 2006). As shown in Figure 1a, upon lysolecithin‐induced demyelination MMP7 mRNA levels were increased at demyelination (3 and 5 DPL), while both MMP3 and MMP7 mRNA levels were significantly enhanced at remyelination (14 DPL). Coincidentally, mRNA levels of TIMP1, a natural inhibitor of MMPs, were substantially increased at demyelination, and decreased at remyelination (14 DPL) compared with 5 DPL (Figure 1a). This indicates that MMP3 and MMP7 may exhibit higher activity at 14 DPL. Western blot (Figure 1b,d) and immunohistochemical (Figure 1e) analyses confirmed the increased expression of MMP7 protein at remyelination (14 DPL) compared with demyelination (5 DPL). A clear shift from proMMP7 to MMP7 was observed at remyelination (Figure 1b,d), and double staining with the microglia/macrophage marker Iba1 demonstrated that (pro)MMP7 is present in microglia/macrophages (Figure 1e, arrow), as well as extracellularly, which may represent secreted (pro)MMP7. The increased level of MMP3 mRNA was not reflected by an increased protein level of proMMP3 and MMP3 at 5 and 14 DPL (Figure 1b,c). Next, the expression of MMP3 and MMP7 upon cuprizone‐induced demyelination was examined. Feeding animals with the copper‐chelator cuprizone for several weeks induces reproducible demyelination of the *corpus callosum*, while withdrawal of cuprizone is followed by remyelination (Gudi, Gingele, Skripuletz, & Stangel, 2014). Upon 3 and 5 weeks of cuprizone treatment, demyelinated areas were readily visible in the *corpus callosum*, while remyelination, as reflected by the reappearance of Sudan Black staining, occurs 2 weeks after cuprizone removal (Figure 2a). Western blot analysis of total *corpus callosum* homogenates demonstrated increased proMMP3 levels during remyelination compared with control and demyelinating conditions (Figure 2b,c), consistent with previous reports (Ulrich et al., 2006; Škuljec et al., 2011). Also, (pro)MMP7 and MMP7 were present during demyelination and remyelination, while in contrast to lysolecithin‐induced demyelination, no difference in their ratio was observed (Figure 2b,d). Immunohistochemical analysis confirmed the similar (pro)MMP7 expression levels (Supporting Information Figure S1). In contrast to lysolecithin‐induced lesions, (pro)MMP7 was scarcely localized to Iba1‐positive microglia/macrophages and appears to localize extracellularly (Supporting Information Figure S1, insets), while a prominent increase in Iba1‐positive microglia/macrophages was evident at 3 and 5 weeks demyelination. This discrepancy may be explained by taking into account that in the lysolecithin‐induced lesions (pro)MMP7 levels were examined during early remyelination, whereas in the cuprizone model (pro)MMP7 levels were analyzed at conditions at which remyelination nears completion. This suggests that microglia/macrophages transiently express MMP7, and therefore, the presence of (pro)MMP7 during remyelination upon cuprizone‐induced demyelination may be underestimated. Hence, these findings show that (pro)MMP3 and (pro)MMP7 are present in demyelinated areas, and that their expression levels are enhanced during the remyelination process, indicating that they may be involved in ECM remodeling upon injury, including fibronectin clearance, which we examined next.

### 3.2 MMP7 cleaves fibronectin aggregates into a 13 kDa EIIIA (rat)/16 kDa EDA (human)‐containing fragment

**Figure 3:**
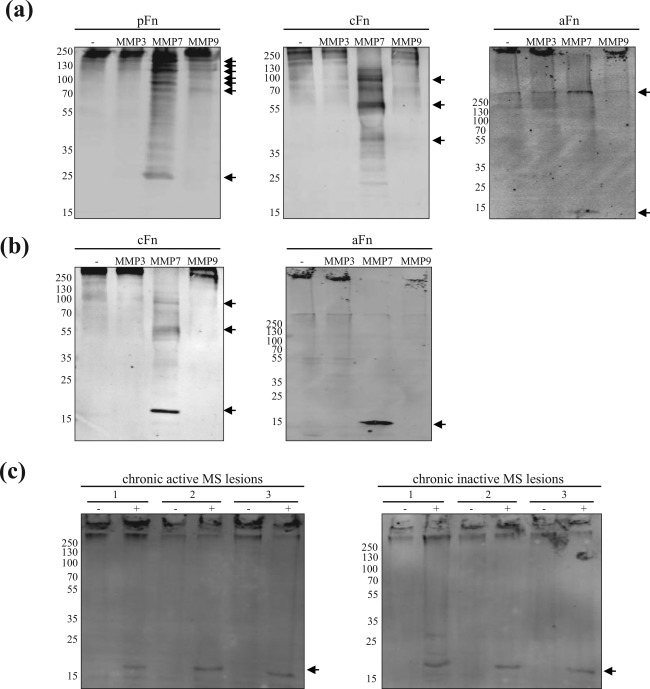
Degradation of fibronectin structural variants by recombinant MMPs. (a and b) Bovine plasma fibronectin (pFn, 5 µg), human cellular fibronectin (cFn, 5 µg) and aggregated rat fibronectin (aFn, 5 µg) were incubated with vehicle (PBS, −), or recombinant human MMP3, MMP7, or MMP9 for 24 hr at 37°C and subjected to Western blotting (non‐reducing) using a polyclonal anti‐Fn antibody (a, total Fn) or an antibody directed against the EIIIA domain of cFn (b, EIIIA/EDA‐Fn). Representative blots of three independent experiments are shown. Note that MMP7 efficiently degrades pFn, cFn, and aFn, while different Mw degradation products of either structural variant are obtained. (c) MMP7‐mediated degradation of human fibronectin aggregates obtained from 100 µg of three distinct chronic active and chronic inactive MS lesions of three different MS patients as assessed with Western blot (non‐reducing, anti‐EIIIA Fn antibody). Arrow indicates main fibronectin degradation products

Both plasma and cellular fibronectin are assembled into fibronectin aggregates (Stoffels et al., 2013). Therefore, to examine the ability of MMPs to digest fibronectin, we incubated different fibronectin structural variants, including fibronectin aggregates, with active human recombinant MMP3, MMP7, and MMP9. When plasma (bovine) and cellular (human) fibronectin were incubated with recombinant MMP7, the protein was degraded, and multiple fragments, as detected by Western blot analysis (Figure 3a), were obtained after 24‐hr incubation. Intriguingly, the molecular weight (Mw) of the generated fibronectin fragments obtained with the distinct structural variants recognized by a polyclonal anti‐fibronectin antibody, differed. MMP7‐mediated degradation of plasma fibronectin generated 6 prominent fragments with a Mw between 154 and 82 kDa, and a fragment with an apparent Mw of 23.5 kDa, while MMP7‐mediated cleavage of cellular fibronectin resulted in fragments of 110, 60 and 28 kDa (Figure 3a, arrows). MMP3 and MMP9 were less efficient in the degradation of dimeric plasma and cellular fibronectin within the 24‐hr time frame. Although MMPs and fibronectin are well‐conserved among species and while human cellular fibronectin is also not degraded, it cannot be excluded that the lack of bovine plasma fibronectin degradation by MMP3 and MMP9 is due to species differences. When aggregated fibronectin was incubated with the recombinant MMPs, an ∼9 kDa fragment appeared as major product following incubation with MMP7, but not with MMP3 and MMP9. Silver staining showed a similar degradation profile of the MMPs, as well as additional bands apart from the recombinant MMPs themselves (data not shown). Fibronectin aggregates were obtained from poly(I:C)‐stimulated astrocytes and consists of both bovine plasma and rat cellular fibronectin. Plasma and cellular fibronectin are distinguished by the sole presence of extradomain EIIIA and/or extradomain EIIIB (EDA and EDB in human) in cellular fibronectin (Paul, Schwarzbauer, Tamkun, & Hynes, 1986). To visualize cellular fibronectin‐derived degradation products, the degradation fragments of cellular and aggregated fibronectin were analyzed with a monoclonal antibody that specifically recognizes the EIIIA/EDA domain. A fragment with an apparent Mw of 13 kDa was clearly visible upon MMP7‐mediated degradation of aggregated fibronectin (Figure 3b), which is similar to the predicted Mw of the full EIIIA domain (Shinde et al., 2008). This suggests that MMP7‐catalyzed cleavage of aggregated fibronectin involves the release of the EIIIA domain. MMP7‐mediated cleavage of human cellular fibronectin generated three major degradation products of approx. 102, 59, and 16 kDa. The 16‐kDa product is likely the EDA domain, that is, the human equivalent of the EIIIA domain (Stine, Sun, Armstrong, Bowler, & Briknarová, 2015). To assess whether MMP7 is able to degrade MS lesion‐derived fibronectin aggregates, aggregates obtained from three chronic active and three chronic inactive MS lesions from different patients were incubated with recombinant MMP7. Using the anti‐EIIIA/EDA‐fibronectin antibody, a 16‐kDa degradation product was visible, which is likely the human equivalent of the 13‐kDa EIIIA‐containing fragment, generated from rat‐derived astrocytes (Figure 3c). In contrast to rat‐derived astrocytes, significant quantities of intact aggregates exist, indicating that MS lesion‐derived aggregates are more stable or reflecting a limited MMP7‐induced cleavage. Together, the data demonstrate that recombinant MMP7 catalysed the cleavage of fibronectin aggregates into a small EIIIA/EDA‐domain containing fragment.

### 3.3 MMP7‐cleaved fibronectin aggregate coatings inhibit OPC maturation

**Figure 4:**
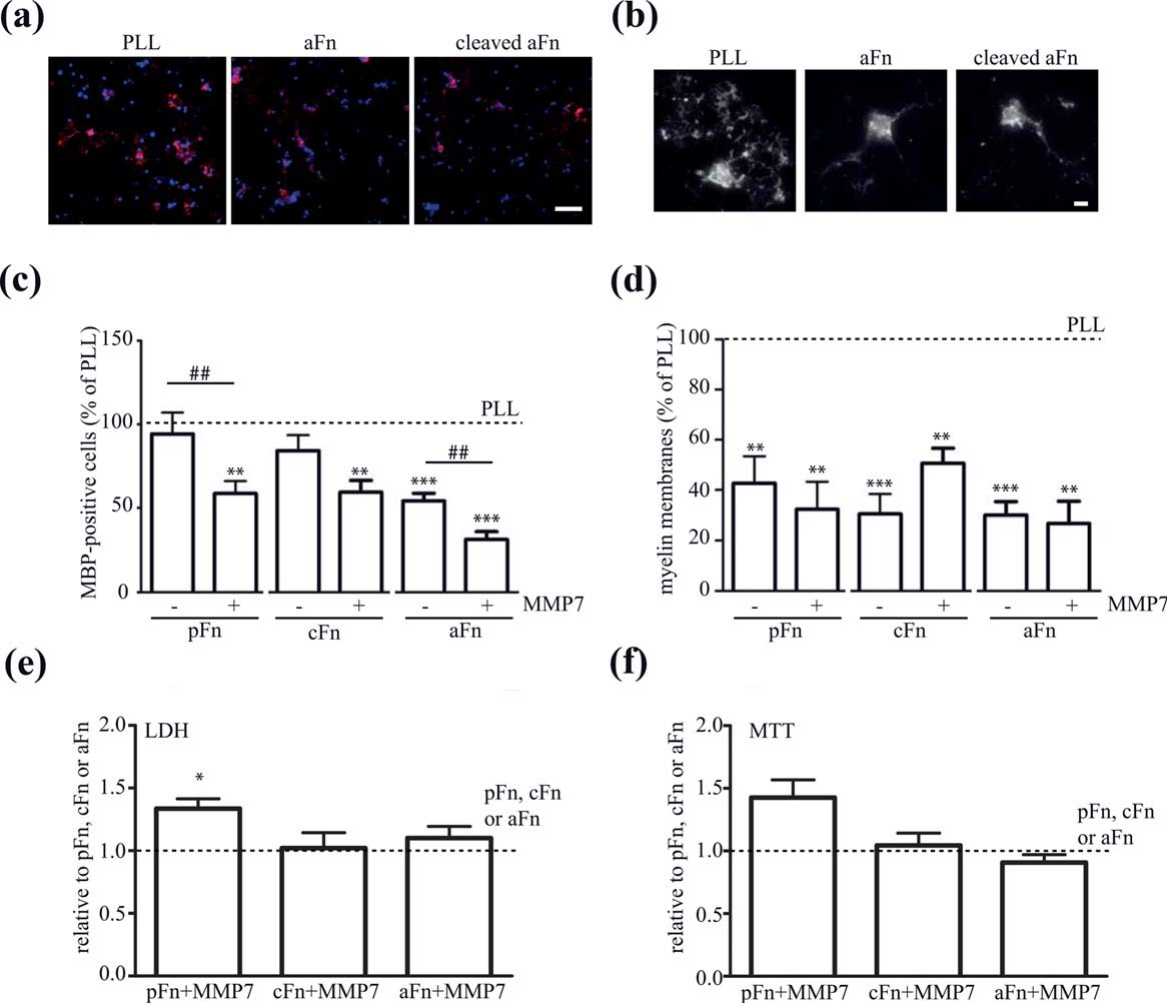
OPC maturation on MMP7‐cleaved plasma fibronectin, cellular fibronectin and fibronectin aggregates. Oligodendrocyte progenitor cells (OPCs) were differentiated on poly‐l‐lysine (PLL), intact plasma fibronectin (pFn), cellular fibronectin (cFn), fibronectin aggregates (aFn) or MMP7‐degraded plasma fibronectin (pFn‐MMP7), cellular Fn (cFn + MMP7) or fibronectin aggregates (aFn‐MMP7) for 6 days. The experiments on the structural variants are performed in independent experiments, while PLL is used as a positive control in each experiment. (a and b) Representative overview (a) and single cell (b) images of MBP immunocytochemistry (red) of oligodendrocytes (OLGs, 6 days in differentiation) cultured on the indicates substrates. DAPI‐stained nuclei are indicated in blue. Scale bar are 75 μm (a) and 10 μm (b). (c and d) The percentage of MBP‐positive cells (c) and the percentage of MBP‐positive cells bearing myelin membranes (d) were assessed 6 days after initiating differentiation. Bars depict mean + *SEM* of at least five independent experiments. In each experiment, the data of cells cultured on PLL was set at 100% (horizontal line). The percentages of MBP‐positive cells or myelin membranes in cells cultured on PLL were respectively 33.6.8% ± 9.5% and 24.0% ± 10.2% for the pFn‐related experiments, 34.9.8% ± 8.6% and 26.1% ± 8.2% for the cFn‐related experiments, and 43.8% ± 11.9% and 29.1% ± 8.7% for the aFn‐related experiments. Statistical differences with cells cultured on PLL (one sample *t* test, ***p < .001, *n* = 5) and intact fibronectin (Student's *t* test, ##*p* < .01) are indicated. Note, that MMP7‐cleaved plasma, cellular and aggregated fibronectin inhibited OPC differentiation compared with their intact counterparts. (e and f) Cytotoxicity (e, LDH release in culture medium) and cell viability (f, MTT reduction) assays of OPCs cultured for 2 days at the indicated substrates. Bars depict mean + *SEM* of four independent experiments. In each experiment, the data of respective intact substrates was set at 100% (horizontal line). Statistical analyses were performed using the one‐sample *t* test when compared with the intact substrate (not significant) [Color figure can be viewed at http://wileyonlinelibrary.com]

To assess whether the MMP7‐mediated proteolytic digestion of fibronectin aggregates is sufficient to overcome the aggregated fibronectin‐mediated inhibition of myelin membrane formation, OPCs were plated on intact fibronectin aggregates or aggregates that were cleaved with recombinant MMP7 (see Figure 3). As shown in Figure 4, MMP7‐cleaved fibronectin aggregate coatings inhibited OPC differentiation as reflected by a decrease in the percentage of MBP‐positive cells compared with cells grown on PLL. In fact, on MMP7‐cleaved fibronectin aggregate coatings the percentage of MBP‐positive cells was reduced compared with cells that were cultured on intact fibronectin aggregates (Figure 4a,c). Consistent with previous findings (Buttery & ffrench‐Constant, 1999, Šišková et al., 2007, 2009, Stoffels et al., 2013), oligodendrocytes plated on fibronectin structural variants are morphologically immature compared with oligodendrocytes plated on PLL (Figure 4b). Similar experiments with MMP7‐cleaved plasma and cellular fibronectin coatings revealed an augmented inhibition of OPC differentiation when plated on MMP7‐cleaved fragments compared with intact fibronectin (Figure 4c). Notably, coatings of the cleaved fibronectin fragments did not contain MMP7 (data not shown), indicating that MMP7 is washed away upon coating and that a potential carry‐over effect of MMP7 can be excluded. This indicates, that fibronectin fragments, either plasma or cellular fibronectin‐derived, more prominently decreased OPC differentiation compared with intact fibronectin. In addition, intact fibronectin aggregate, and to a lesser extent cellular fibronectin, but not plasma fibronectin coatings, inhibited OPC differentiation compared with PLL (Figure 4c). We have previously shown that aggregated fibronectin is not toxic to oligodendrocytes (Stoffels et al., 2013). To exclude that MMP7‐cleaved fibronectin fragments were toxic and/or affected the viability of cells, we performed LDH and MTT assays. Coatings of MMP7‐cleaved cellular fibronectin and aggregated fibronectin fragments scarcely affected the release of LDH (Figure 4e) or levels of MTT reduction (Figure 4f) compared with their intact counterparts, while MMP7‐cleaved plasma fibronectin coatings were slightly toxic. However, the concomitant increase rather than decrease in MTT reduction, suggests an increase in the number of cells on an MMP7‐cleaved plasma fibronectin coating, which is likely due to an increase in OPC proliferation rather than cell death. MMP7‐cleaved fibronectin aggregate, cellular fibronectin and plasma fibronectin coatings inhibited myelin membrane formation compared with PLL to a similar extent as their intact counterparts, as indicated with the similar percentage of cells that extended MBP‐positive myelin membranes of total MBP positive cells (Figure 4d). Hence, MMP7‐mediated cleavage of fibronectin (aggregates) is a first step to remove fibronectin (aggregates), but as the remaining degradation products inhibit OPC differentiation, further clearance by, for example, astrocytes, microglia or macrophage‐mediated phagocytosis and/or other proteases is required.

### 3.4 Levels of proMMP7 are reduced in chronic active and chronic active MS lesions

**Figure 5:**
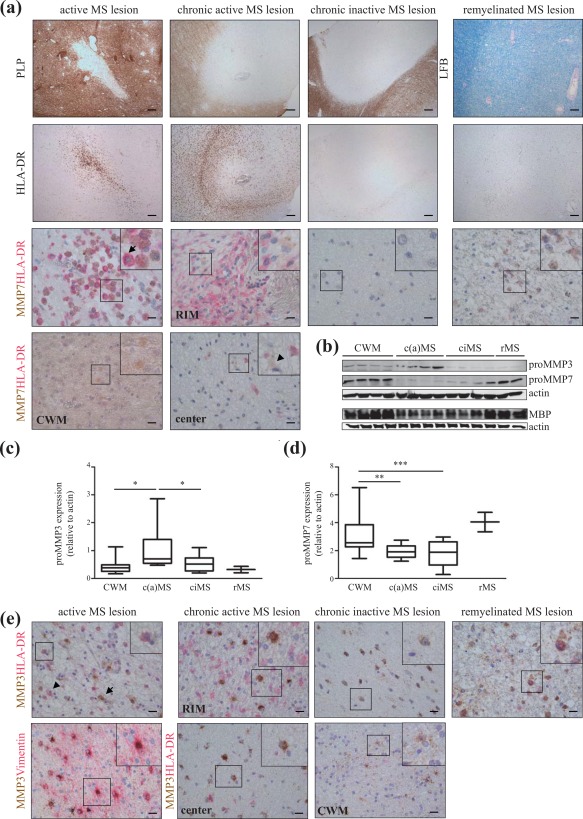
MMP protein localization and expression in control white matter and MS lesions. (a) Serial cryosections of post‐mortem control white matter (*n* = 3), active MS lesions (*n* = 3), chronic active MS lesions (*n* = 3), chronic inactive MS lesions (*n* = 3) and remyelinated MS (*n* = 3) lesions of distinct MS patients were stained for PLP or luxol fast blue (LFB), and MHC II (HLA‐DR), and double‐stained for HLA‐DR and MMP7. Lesion activity was characterized by HLA‐DR and PLP expression; remyelinated lesions were characterized by LFB. Arrow indicates a HLA‐DR and MMP7 positive microglia/macrophage; arrowhead indicates a cell that morphologically resembles astrocytes. Scale bars are 20 (double stainings) or 200 μm (single staining). Note that in active and remyelinated MS lesions MMP7 is clearly present in microglia/macrophages. (b) Protein levels of (pro)MMP3 and (pro)MMP7 in brain white matter homogenates (50 µg) of human control white matter (CWM, *n* = 9), (chronic) active [(c)aMS, *n* = 8], chronic inactive (ciMS, *n* = 9) and remyelinated (rMS, *n* = 2) MS lesions as determined by Western blotting. Demyelination was confirmed by the decreased MBP expression. Actin serves as loading control. (c and d) Quantification of proMMP3 (c) and proMMP7 (d). Statistical differences were assessed with a one‐way ANOVA (*p < .05, Newman‐Keuls, *n* ≥ 8). Note the decreased levels of proMMP7 in MS lesions compared with CWM, and the slight increase of proMMP3 expression in (chronic) active MS lesions. (e) Serial cryosections of (a) double‐stained for HLA‐DR and MMP3 or vimentin (astrocyte marker) and MMP3. Arrow indicates a HLA‐DR‐positive and (pro)MMP3‐negative microglia/macrophage; arrowhead indicates a cell that morphologically resembles astrocytes. Note the prominent presence of (pro)MMP3 in vimentin‐positive astrocytes in (chronic) active lesions. Scale bars are 20 μm [Color figure can be viewed at http://wileyonlinelibrary.com]

Although enhanced presence of MMP7 in active demyelinating MS lesions is well documented (Anthony et al., 1997; Cossins et al., 1997; Lindberg et al. 2001), the persistence of fibronectin (aggregates) in chronic MS lesions suggests that the inability to clear fibronectin is due to the lack of expression and/or impairment of its activity. Therefore, we next analyzed the presence of MMP7 in post‐mortem MS and control tissue. Of note, the anti‐MMP7 antibody does not distinguish proMMP7 from MMP7 (Figures 1b and 2b). In control tissue, (pro)MMP7 is diffusely expressed throughout the white matter (Figure 5a). In active MS lesions, characterized by HLA‐DR‐expressing microglia/macrophages in the demyelinated area (Figure 5a, van der Valk & De Groot, 2000), (pro)MMP7 localized to HLA‐DR‐positive microglia/macrophages (Figure 5a, arrow), corroborating previous studies (Anthony et al., 1997; Cossins et al., 1997). Co‐stainings of MMP7 with human phenotype‐specific differentially‐activated microglia/macrophages markers (Peferoen et al., 2015) in active MS lesions showed that (pro)MMP7 was not restricted to and only occasionally present in both classical CD40‐positive and pro‐regenerative CD206‐positive microglia/macrophages (Supporting Information Figure S2a,b). In chronic active MS lesions, characterised by a hypocellular lesion centre and a rim of HLA‐DR‐positive microglia/macrophages (Figure 5a), (pro)MMP7 is less abundantly expressed in the rim and lesion center compared with active lesions. In addition, (pro)MMP7 is occasionally present in cells with an astrocyte‐like morphology (Figure 5a, arrowhead). This occasional presence of (pro)MMP7 in astrocytes was confirmed by double labeling with the activated astrocyte marker vimentin (Supporting Information Figure S2c). In chronic inactive MS lesions, which lack HLA‐DR‐positive microglia/macrophages, (pro)MMP7 immunoreactivity is absent or only weakly expressed. Remarkably, in remyelinated MS lesions, identified as a myelinated area with a less intense LFB staining (Figure 5a), HLA‐DR‐positive macrophages harbor (pro)MMP7 immunoreactivity, while also a more diffuse immunoreactivity of (pro)MMP7 as in CWM was observed. To confirm this differential expression, we next analyzed MMP7 expression levels in chronic active and chronic inactive MS lesions homogenates, previously shown to contain fibronectin aggregates (Stoffels et al., 2013) by Western blotting. While present in CWM and remyelinated lesions, proMMP7 levels were significantly reduced in homogenates of chronic active and inactive MS lesions (Figure 5b,d). In contrast, proMMP3, in its active form known to be an activator of MMP7 (Imai et al., 1995), was slightly increased in chronic active MS lesion homogenates compared with CWM and chronic inactive MS lesion homogenates (Figure 5b,c). Immunohistochemical analysis revealed that (pro)MMP3 was only occasionally present in HLA‐DR‐positive microglia/macrophages in active and remyelinated MS lesions, whereas (pro)MMP3 was prominently present in vimentin‐positive astrocytes (Figure 5e). Hence, these findings indicate that the absence of sufficient levels of (pro)MMP7 in chronic active and inactive MS lesions correlates to the persistent presence of fibronectin (aggregates). The decreased (pro)MMP7 expression in chronic MS lesions may be a result of inappropriate or impaired activation of cells that produce MMP7 upon myelin injury. Therefore, we next examined *in vitro* which type(s) of resident cells, present in MS lesions, is (are) the major source(s) of these MMPs.

### 3.5 IL‐4‐activated microglia and macrophages are a major cellular source of secreted proMMP7

**Figure 6:**
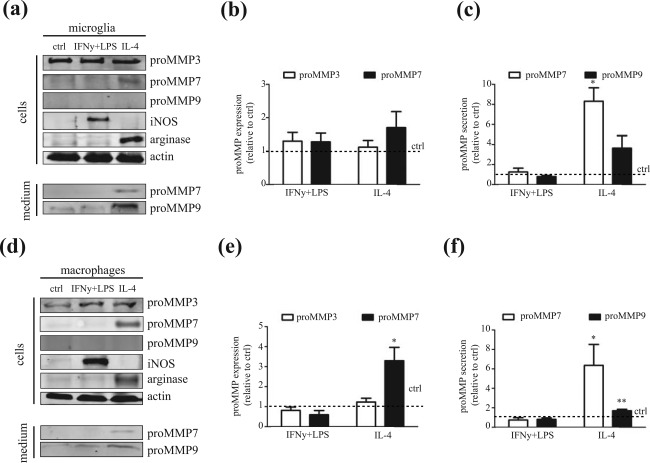
MMP expression in microglia and bone‐marrow derived macrophages. (a–c) Protein levels of (pro)MMP3, (pro)MMP7, and (pro)MMP9 in control, IFNγ+LPS‐activated and IL‐4‐activated microglia in total cell lysates (50 µg) and cell‐conditioned medium (CM, 40 µl) as determined by Western blot. Representative blots of three to eight independent experiments (a), quantitative analysis of proMMP3 and proMMP7 in cell lysates (b), and proMMP7 and proMMP9 levels in medium (c). Actin serves as a loading control. (d–f) Protein levels of MMP3, MMP7, and MMP9 in control, IFNγ+LPS‐activated and IL‐4‐activated bone marrow‐derived macrophages in total cell lysates (50 µg) and cell‐conditioned medium (CM, 40 µl) as determined by Western blot. Representative blots of six to fifteen independent experiments (d), quantitative analysis of proMMP3 and proMMP7 in cell lysates (e), and proMMP7 and proMMP9 levels in medium (f) are shown. Actin serves as a loading control. Bars depict mean + *SEM* relative to control. Statistical differences as assessed with a one‐sample *t* test are indicated (*p < .05, *n* ≥ 4)

Several *in vitro* and *in vivo* studies reveal that resident microglia and astrocytes, as well as infiltrating macrophages produce MMPs (Anthony et al., 1997; Cossins et al., 1997; Škuljec et al., 2011). To identify cellular sources of (pro)MMP7 expression and release, and to explain its impaired expression in chronic MS lesions, we examined the presence MMP7 in lysates and in conditioned medium of cultured microglia and astrocytes, as well as bone marrow‐derived macrophages (referred to as “macrophages”) under resting (control) and/or MS‐relevant conditions that do not affect cell viability. In addition, given their role in MS pathology and/or their ability to activate MMP7, we also examined cellular sources of (pro)MMP3 and (pro)MMP9. Models of microglia and macrophage activation are often simplified to classical activation, evoked by exposure to IFNγ and LPS, or alternative activation, generated by exposure to IL‐4. These phenotypes are regarded as the two extremes of a continuum of microglia/macrophage activation states (Wolf, Boddeke, & Kettenmann, 2017; Murray, 2017). As shown in Figure 6a–c, the expression levels of proMMP3 and proMMP7 in cell lysates of IFNγ+LPS‐activated and IL‐4‐activated microglia were similar, while proMMP7 and proMMP9 levels were enhanced in conditioned medium of IL‐4‐activated compared with IFNγ+LPS‐activated and non‐stimulated control microglia. ProMMP3 was not detectable in conditioned microglia medium (data not shown). IL‐4‐activated macrophages showed a significant increase in proMMP7 levels, while proMMP3 levels were similar at all conditions (Figure 6d–f). As in microglia, proMMP9 was weakly detectable in lysates of macrophages. Similar to microglia, proMMP7 and proMMP9 levels were increased in conditioned medium of IL‐4‐activated macrophages (Figure 6d,f), whereas proMMP3 was hardly detectable (data not shown). Both (active) MMP7 and MMP9 were hardly present in microglia and macrophages lysates and conditioned medium. Importantly, consistent with the corresponding phenotype, iNOS expression was increased upon exposure to IFNγ+LPS, and arginase‐1, a marker for alternative activated microglia and macrophages, was expressed upon IL‐4 treatment (Figure 6a,d). Pro‐inflammatory‐activated astrocytes, a condition that is relevant to MS lesions (Nair, Frederick, & Miller, 2008; Sofroniew & Vinters, 2010), showed increased levels of MMP7, but not proMMP3 and proMMP7 compared with control astrocytes (Supporting Information Figure S3). ProMMP7 and proMMP9, but not MMP7, were detected in pro‐inflammatory‐activated astrocyte conditioned medium, while the MMPs were virtually absent in conditioned medium of control astrocytes (Supporting Information Figure S3). While OPCs are present in most MS lesions, their differentiation seems to be inhibited (Lucchinetti et al., 1999, Kuhlmann et al., 2008, Chang et al., 2012), which may account for the observed alterations in MMP expression levels. Upon differentiation of OPCs to myelinating oligodendrocytes, proMMP3 expression significantly increased, while proMMP7 and proMMP9 were hardly detected at either differentiation stage (Supporting Information Figure S3). ProMMP3 was virtually absent in conditioned medium of primary oligodendrocyte cultures (data not shown). Hence, pro‐inflammatory cytokine‐activated astrocytes, and IL‐4‐activated microglia and macrophages secrete proMMP7 levels that may potentially degrade fibronectin aggregates.

### 3.6 MMP‐activated conditioned medium of IL‐4‐activated microglia and macrophages cleaves fibronectin aggregates

**Figure 7:**
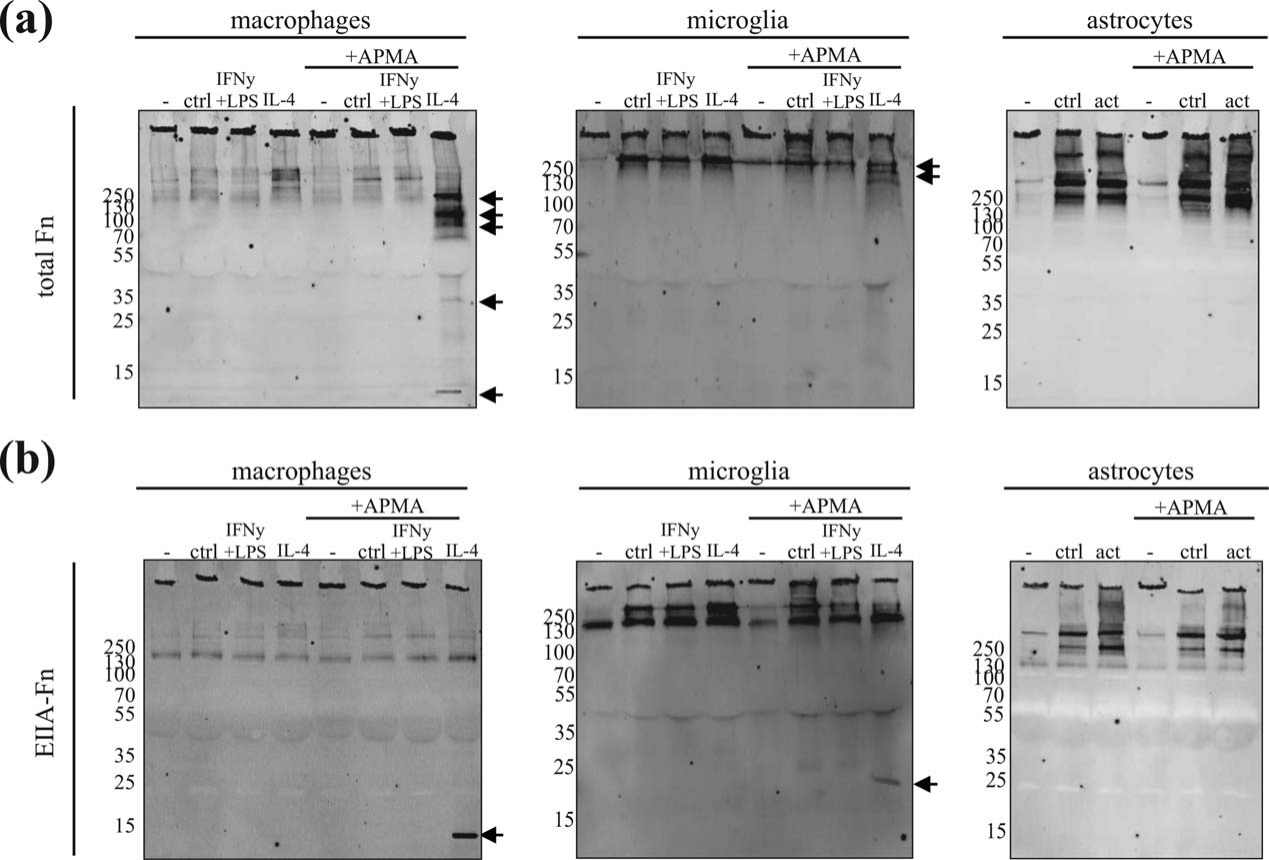
Degradation of aggregated fibronectin by conditioned medium. (a and b) Aggregated fibronectin (aFn, 5 µg) was incubated with non‐conditioned (–) or conditioned medium of control (ctrl), and IFNγ+LPS− and IL‐4‐actived bone marrow‐derived macrophages and microglia, or control and pro‐inflammatory cytokine (TNFα, IFNγ, and IL‐1β) activated astrocytes for 72 hr at 37°C and subjected to Western blotting (non‐reducing) using a polyclonal fibronectin (total Fn, a) or anti‐EIIIA‐fibronectin (EIIIA‐Fn, b) antibody. MMPs present in medium are not activated or activated by the general MMP activator APMA. Representative blots of three independent experiments are shown. Arrow indicates main fibronectin degradation products

As IL‐4‐activated microglia, macrophages, and cytokine‐activated astrocytes secreted significant proMMP7 levels, we next determined whether MMPs present in the conditioned medium of either cell type were sufficient to cleave aggregated fibronectin. Incubation with aggregated fibronectin for 72 hr at 37°C showed that conditioned medium of either cell type was insufficient in cleaving aggregated fibronectin (Figure 7). As Western blot analysis revealed that MMP7 was present as proenzyme (Figure 6), we treated the conditioned medium with APMA, a general activator of MMPs. Interestingly, APMA‐treated, that is, MMP‐activated, conditioned medium from IL‐4‐activated macrophages, and to a lesser extent microglia cleaved aggregated fibronectin (Figure 7a,b). In IL‐4‐activated macrophages and microglia, similar to recombinant MMP7‐mediated cleavage of aggregated fibronectin, a major degradation product visualized with the anti‐EIIIA fibronectin antibody was a 13 kDa fragment (Figure 7b cf Figure 3b). Also, the 9 kDa product that appears upon recombinant MMP7‐cleavage of aggregated fibronectin using the polyclonal fibronectin antibody was visible (Figure 7a cf Figure 3a), indicating that MMP7 present in macrophage‐conditioned medium likely cleaved the aggregated fibronectin. In addition, and in contrast to cleavage with recombinant MMP7, prominent fragments at 164, 103, 88, and 32 kDa were visualized with the polyclonal anti‐fibronectin antibody. This may relate to the presence of macrophage‐secreted fibronectin in the conditioned medium. Macrophages and astrocytes, and to a lesser extent microglia secreted pronounced levels of fibronectin (Supporting Information Figure S4). However, these fragments and the 13 kDa fragment were not detected upon MMP‐activation (APMA) of conditioned medium only (Supporting Information Figure S4), indicating that these degradation fragments were derived from aggregated fibronectin and not from cellular fibronectin. Of note, conditioned medium was made with plasma fibronectin‐free serum. Complete MMP‐mediated degradation of the aggregates was not observed indicating that cleavage of aggregated fibronectin with IL‐4‐activated macrophage conditioned medium was less efficient than recombinant MMP7. Notably, with the polyclonal anti‐fibronectin antibody different fibronectin degradation products (160 and 151 kDa) were visualized with IL‐4‐activated microglia‐conditioned medium but not in IL‐4‐activated macrophage‐conditioned medium (Figure 7a). To examine whether fibronectin‐degrading proteinases other than MMP7 and MMP9, for example, other metalloproteinases (MMPs, ADAMTS) and serine proteinases (plasmin), were expressed and potentially secreted by these cells and that may explain the difference with the recombinant MMP7‐degradation profile, a comprehensive RT‐qPCR analyses was performed. In addition, the mRNA levels of TIMPs, which are natural inhibitors of MMPs, were determined. As shown in Table 3, MMP3, MMP13, ADAMST4 (only macrophages) and TIMP1 (only macrophages) were enhanced in IFNγ+LPS‐activated microglia and/or macrophages, and consistent with the Western blot data (Figure 6) only MMP7 mRNA was enhanced upon IL‐4 treatment. This further supports the likelihood of MMP7 being secreted by IL‐4‐activated microglia and macrophages that cleaved aggregated fibronectin.

**Table 3:** RT‐qPCR analysis of primary microglia, bone marrow‐derived macrophages and astrocytes

	Microglia		Macrophages		Astrocytes
	IFNγ+LPS	IL‐4	IFNγ+LPS	IL‐4	TNFα+IFNγ+IL1β
*MMP3*	5.34 ± 1.25*	0.76 ± 0.06^***^	9.16 ± 2.56^*^	**0.76 ± 0.12**	25.36 ± 6.78^***^
*MMP2*	1.09 ± 0.19	0.98 ± 0.10	0.85 ± 0.06	0.89 ± 0.10	1.61 ± 0.37
*MMP7*	0.52 ± 0.16^*^	4.45 ± 0.90^*^	0.27 ± 0.04^***^	3.17 ± 0.38^***^	2.96 ± 1.46
*MMP8*	1.43 ± 0.69	0.67 ± 0.37	0.27 ± 0.12^***^	0.65 ± 0.21	1.93 ± 0.41
*MMP9*	1.57 ± 0.31	1.34 ± 0.23	1.06 ± 0.16	1.04 ± 0.12	15.87 ± 5.72^*^
*MMP11*	0.72 ± 0.14	0.87 ± 0.10	1.02 ± 0.22	1.18 ± 0.13	0.72 ± 0.22
*MMP12*	1.03 ± 0.28	1.05 ± 0.12	0.66 ± 0.16	1.10 ± 0.12	17.67 ± 5.52^*^
*MMP13*	4.31 ± 0.94^*^	0.96 ± 0.11	2.44 ± 0.53^*^	0.90 ± 0.14	16.14 ± 4.57^*^
*MMP14*	1.16 ± 0.20	0.59 ± 0.10^*^	1.05 ± 0.18	0.54 ± 0.10^***^	0.87 ± 0.13
*ADAMST4*	1.21 ± 0.40	0.96 ± 0.07	3.88 ± 0.77^*^	0.67 ± 0.12^*^	n.d.
*TIMP1*	1.88 ± 0.41	1.25 ± 0.13	3.00 ± 0.30^***^	1.00 ± 0.08	2.92 ± 0.38^***^
*TIMP2*	0.60 ± 0.05^*^	1.16 ± 0.07	0.52 ± 0.11^*^	1.11 ± 0.03^*^	1.24 ± 0.27
*TIMP3*	1.45 ± 0.46	0.78 ± 0.10	1.47 ± 0.15^*^	1.00 ± 0.22	2.27 ± 0.42^*^
*TIMP4*	0.91 ± 0.16	1.19 ± 0.12	0.58 ± 0.15	1.24 ± 0.15	n.d.
*tPA*	4.2 ± 1.60	1.51 ± 0.30	1.80 ± 0.36	0.72 ± 0.05^*^	2.07 ± 0.21^***^
*uPA*	3.51 ± 2.19	2.00 ± 1.00	0.59 ± 0.43	0.82 ± 0.08	3.62 ± 1.68

All data relative to (untreated) control are shown, which was set to 1 at each independent experiment; Data are expressed as mean ± SEM (*n* ≥ 4); Statistical differences with control as assessed with a one sample *t* test are indicated (in bold, *p < .05. ***p < .001).

RT‐qPCR = real‐time quantitative PCR.

Upon MMP‐activating conditions, conditioned medium of cytokine‐activated astrocytes, known to contain proMMP7 and proMMP9 (Supporting Information Figure S3), was not able to fragment aggregated fibronectin (Figure 7a,b). This indicates that other factors present in astrocyte‐conditioned medium may prevent MMP7‐mediated cleavage, or that expression levels of MMPs are too low. Expression profiling showed that upon pro‐inflammatory cytokine exposure, next to transcripts of MMP3, MMP7, MMP9, MMP12, MMP13, and ADAMST, also the natural inhibitors of MMPs, TIMP1, and TIMP3, were increased compared with “resting” astrocytes (Table 3). Hence, macrophages and microglia are potential cellular sources to degrade fibronectin aggregates, and once secreted by IL‐4‐activated microglia/macrophages, MMPs, most likely MMP7, require an appropriate, extracellular activation, for example by other cellular sources, to cleave aggregated fibronectin.

## 4 DISCUSSION

Fibronectin aggregates present in chronic MS lesions impair OPC maturation, and contribute to remyelination failure (Stoffels et al., 2013). Here, we demonstrated that MMP7, cleaved aggregated fibronectin, releasing an EIIIA/EDA‐containing 13 (rat)/16 (human) kDa fragment. ProMMP7 levels were reduced in chronic active and chronic inactive MS lesions compared with control white matter and remyelinated MS lesions, which may explain the persistence of fibronectin aggregates in demyelinated MS lesions. Alternative (IL‐4‐)activated microglia and macrophages expressed and secreted proMMP7, and cleaved fibronectin aggregates *in vitro* at proper MMP‐activating conditions. Hence, given that microglia/macrophage activation is perturbed in MS lesions (Vogel et al., 2013; Peferoen et al., 2015), upregulation of local MMP7 expression levels, for example by priming microglia/macrophages towards the alternative activated phenotype, may be an attractive approach to locally provoke degradation of fibronectin (aggregates) and thus promote remyelination in MS.

Immunohistochemistry and *in situ* hybridization have shown that several fibronectin‐degrading MMPs, including MMP3, MMP7, and MMP9 are upregulated in active demyelinating lesions (Maeda & Sobel, 1996; Anthony et al., 1997; Cossins et al., 1997; Lindberg et al., 2001). These MMPs are present at the rim of active lesions, and less so or confined to perivascular cuffs, in chronic MS lesions (Maeda & Sobel, 1996; Lindberg et al., 2001). The upregulated MMPs are likely part of the neuroinflammatory response that also encompasses disruption of the blood–brain barrier, allowing entry of blood‐derived cells (Rosenberg, Estrada, Dencoff, & Stetler‐Stevenson, 1995; Buhler et al., 2009). However, MMPs also have beneficial roles for remyelination, including ECM remodeling, degradation of myelin debris, and regulation of outgrowth of oligodendrocyte processes (Chandler et al., 1995; Oh et al., 1999; Rosenberg, 2002b; Larsen & Yong, 2004; Šišková et al., 2009). Our present findings show that MMP7 cleaved fibronectin aggregates into small, EIIIA/EDA containing fragments. Previous studies revealed that MMP7 is present in macrophages in active demyelinating lesions (Anthony et al., 1997; Cossins et al., 1997). Our findings confirmed the presence of (pro)MMP7 in activated microglia/macrophages in active MS lesions, and in addition immunohistochemical and Western blot analysis revealed that MMP7 expression was reduced in chronic active and inactive lesions, that is, where fibronectin aggregates are prominently present, compared with control white matter and remyelinated MS lesions. In contrast, MMP3, known to activate MMP7, was increased in chronic active MS lesions and prominently present in astrocytes. Therefore, the inability to clear fibronectin aggregates in chronic MS lesions is likely attributed to the lack of an appropriate protease, for example, MMP7, rather than to the inability to clear dimeric fibronectin. Whether MMP7 degrades fibronectin aggregates *in vivo* remains to be determined.

MMP7 is the smallest MMP, and is particularly upregulated upon neuroinflammation in microglia/macrophages, that is in active demyelinating MS lesions, and in EAE, an animal model of MS (Anthony et al., 1997; Clements et al., 1997; Cossins et al., 1997; Kieseier et al., 1998; Lindberg et al., 2001). MMP7 is implicated in blood–brain barrier disruption, axonal injury and is involved in shedding of signaling molecules, including TNF, that contribute to the pathology of MS (Chandler et al., 1995; Gearing et al., 1995; Kieseier et al., 1998; Newman et al., 2001; Buhler et al., 2009). In contrast, in the present work, we demonstrated that upon lysolecithin‐induced demyelination, MMP7 was upregulated during remyelination, and also present in remyelinated MS lesions. Furthermore, others have shown that MMP7 levels also remain elevated during the remission phase of EAE (Kieseier et al., 1998). As MMP7 efficiently degrades CSPGs and fibronectin, both known to inhibit OPC maturation and transiently expressed upon demyelination while being persistent in MS lesions (Lau et al., 2012; Stoffels et al., 2013), MMP7 may aid to their timely degradation. In addition, since MMP7 is able to degrade MBP (Chandler et al., 1995), this proteinase may aid clearance of remyelination‐inhibiting myelin debris. Nevertheless, MMP7 expression is not noticeably enhanced upon cuprizone‐induced demyelination, consistent with previous findings at the mRNA level (Škuljec et al., 2011). Similar to Škuljec et al., we observed increased expression of proMMP3 upon remyelination in the cuprizone model. Hence, in addition to its detrimental effect in MS lesions, a second local wave of MMP7 activity may be beneficiary by aiding remyelination by clearing OPC maturation‐inhibiting ECM molecules and degrading MBP in myelin debris. Of interest, is that pericellular MMP7‐mediated degradation of fibronectin is accelerated by cells that express sulfatide on their surface (Yamamoto, Miyazaki, & Higashi, 2014). Given that late‐stage OPCs and mature oligodendrocytes are highly enriched in surface sulfatide this may augment local fibronectin degradation.

The enhanced MMP7 mRNA and protein expression upon neuroinflammatory conditions and lysolecithin‐induced demyelination, which is not evident upon cuprizone‐induced demyelination, suggests that MMP7 expression depends on the injury‐mediated activation of local cells. Indeed, expression of MMPs is regulated by a variety of biologically active agents, including growth factors, hormones and cytokines (Clark, Swingler, Sampieri, & Edwards, 2008). Our results indicate that alternative (IL‐4‐)activated microglia and macrophages, but not classical (IFNγ+LPS‐)activated microglia and macrophages, secreted proMMP7 and proMMP9, but not proMMP3. Fibronectin degradation assays demonstrated that alternative (IL‐4‐)activated macrophages and to lesser extent microglia degraded fibronectin aggregates upon MMP‐activating conditions. This indicates that cells other than macrophages and microglia are required to properly activate secreted proMMPs. For example, proMMP7 is activated by MMP3 (Imai et al., 1995), which is highly expressed by astrocytes upon demyelination (Škuljec et al., 2011). Yet, although the investigated cell types expressed proMMP3 *in vitro*, and proMMP3 was particularly upregulated upon OPC maturation, proMMP3 was hardly, if at all present in conditioned medium of either cell type. Also, a central role for plasmin in the activation cascade of MMPs is well documented (Lu et al., 2011), and may be a rate limiting step in proper MMP7 activation. Furthermore, while proMMP7 was secreted by pro‐inflammatory cytokine‐activated astrocytes, fibronectin aggregates remained virtually intact upon incubation with astrocyte conditioned medium at MMP‐activating conditions. Our RT‐qPCR analysis showed that pro‐inflammatory cytokines increased TIMP1 and TIMP3 mRNA expression in astrocytes suggesting that such inhibitory molecules may attenuate MMP activity, adding complexity to regulation of local MMP7 activity.

The decreased MMP7 levels in chronic active and inactive MS lesions may be due to dysfunctional activation of microglia and macrophages. Indeed, microglia and macrophages in inflammatory MS lesions have an intermediate activation status, expressing classical and alternative activated phenotypes (Vogel et al., 2013; Peferoen et al., 2015). The presence of alternative activated microglia/macrophages is essential for remyelination (Miron et al., 2013), and this may rely on appropriate ECM remodeling (Agrawal et al., 2013). Hence, therapeutically inducing local MMP7 expression in microglia/macrophages may be an attractive approach to promote remyelination by resident OPCs in MS lesions. Intriguingly, in other cell types in addition to IL‐4, other inflammatory mediators, including IL‐1, IL‐6, TGFβ, and TNFα, have been implicated in the regulation of MMP7 expression (Klein et al., 1997; Rosenberg, 2002ab; Kato, Haro, Komori, and Shinomiya, 2004; Nagorsen et al., 2005; Clark et al., 2008; Krstic & Santibanez, 2014).

MMP7‐mediated cleavage of fibronectin aggregates was not sufficient to overcome aggregate‐induced inhibition of OPC maturation but rather differentiation was decreased when OPCs were plated on MMP7‐treated fibronectin aggregate coatings, compared with intact fibronectin aggregate coatings. This may be due to exposure of OPCs to EIIIA/EDA‐containing fragments, released upon MMP7‐induced cleavage of the aggregates. The EIIIA/EDA domain, particularly when it is present as a fragment, acts as an endogenous ligand for TLR4 (Okamura et al., 2001; Bhattacharyya et al., 2014). However, OPCs do not express TLR4 (Lehnardt et al., 2003; Sloane et al., 2010; Bsibsi et al., 2012), suggesting that upon release of EIIIA/EDA‐containing fragment the remaining aggregate may be remodeled, which altered the binding and signalling activity to OPCs in a different fashion. Also, MMP7‐treated EIIIA‐lacking plasma fibronectin coatings inhibited OPC differentiation compared with intact plasma fibronectin, corroborating that other fragments than an EIIIA‐containing fragment inhibit OPC differentiation. Therefore, the remaining aggregates may require further processing and clearance by other proteases, e.g., MMP12 (Škuljec et al., 2011), and/or phagocytosis. It is of particular interest in this regard that fibronectin fragments, including EIIIA/EDA itself, can activate MMP expression (Saito et al., 1999; Yasuda et al., 2003).

Thus, local addition, and specific and timely activation of MMP7 may be a therapeutic option to clear remyelination‐inhibiting fibronectin aggregates from MS lesions. However, due to potential side effects such as MMP7‐induced cleavage of (beneficial) laminin (Rosenberg, 2002a; Lu et al., 2011), MMP7‐mediated activation of harmful cytokines (Gearing et al., 1995; Yamamoto et al., 2014), or its toxicity to axons (Newman et al., 2001), such an approach will require careful targeting and timing of MMP7 activation. Accordingly, further knowledge as to why the cleavage pattern of aggregated fibronectin differs from that of plasma fibronectin, as well as identification of MMP7 cleavage sites in the aggregates will be key to induce MMP7 to specifically cleave aggregated fibronectin and enhance remyelination.

## CONFLICTS OF INTEREST

The authors declare no conflict of interest.

## Supporting information

Additional Supporting Information may be found online in the supporting information tab for this article.

Supplementary Figure 1

Supplementary Figure 2

Supplementary Figure 3

Supplementary Figure 4
