# Supplementary figures and images for: MMP7 cleaves remyelination‐impairing fibronectin aggregates and its expression is reduced in chronic multiple sclerosis lesions
Source: Glia. 2018 Mar 30;66(8):1625–43. doi: 10.1002/glia.23328 (PMC6099312; doi:10.1002/glia.23328)

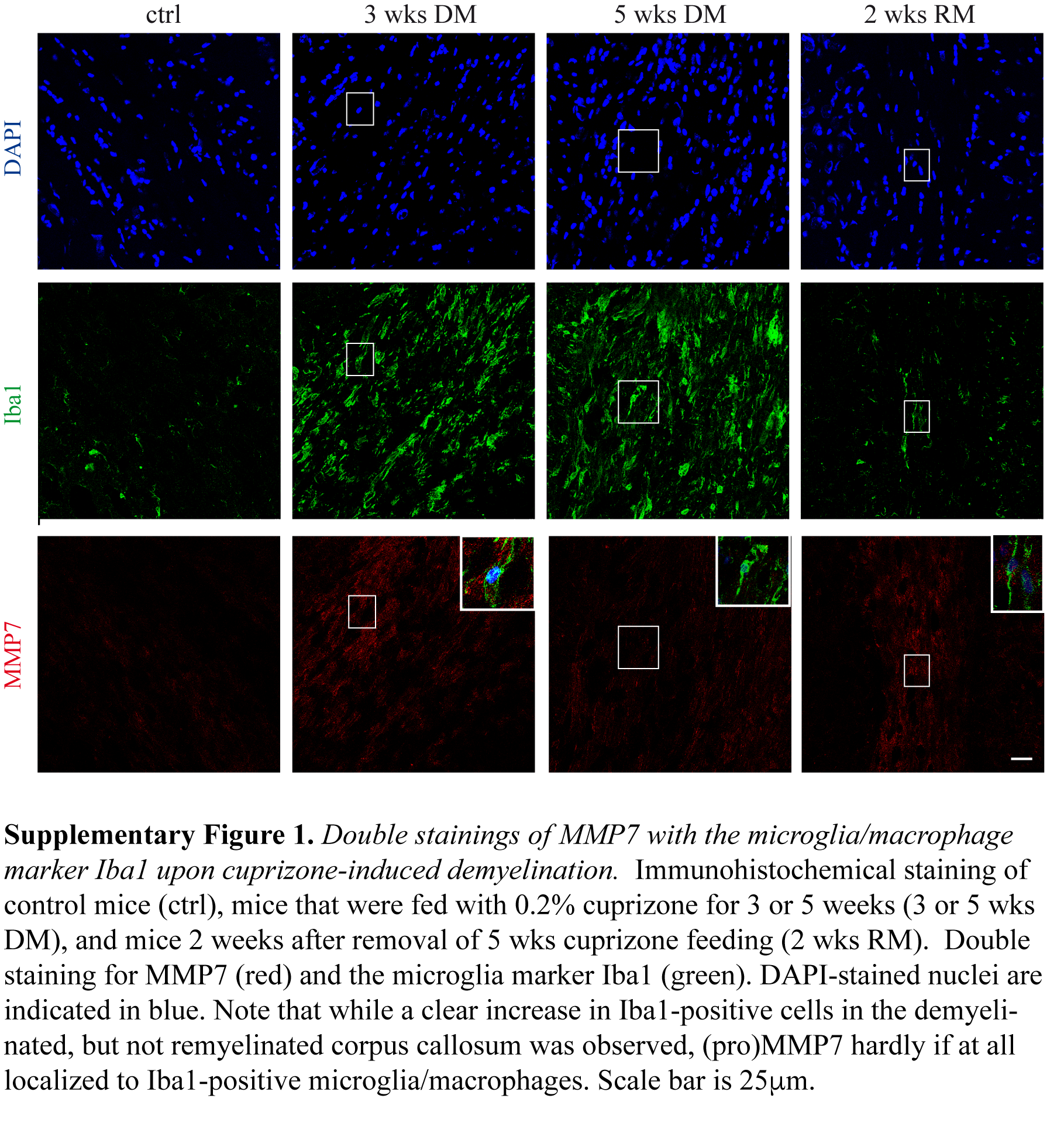

Supplement: Supplementary file 1 — Supplementary Figure 1 [file GLIA-66-1625-s001.tif]

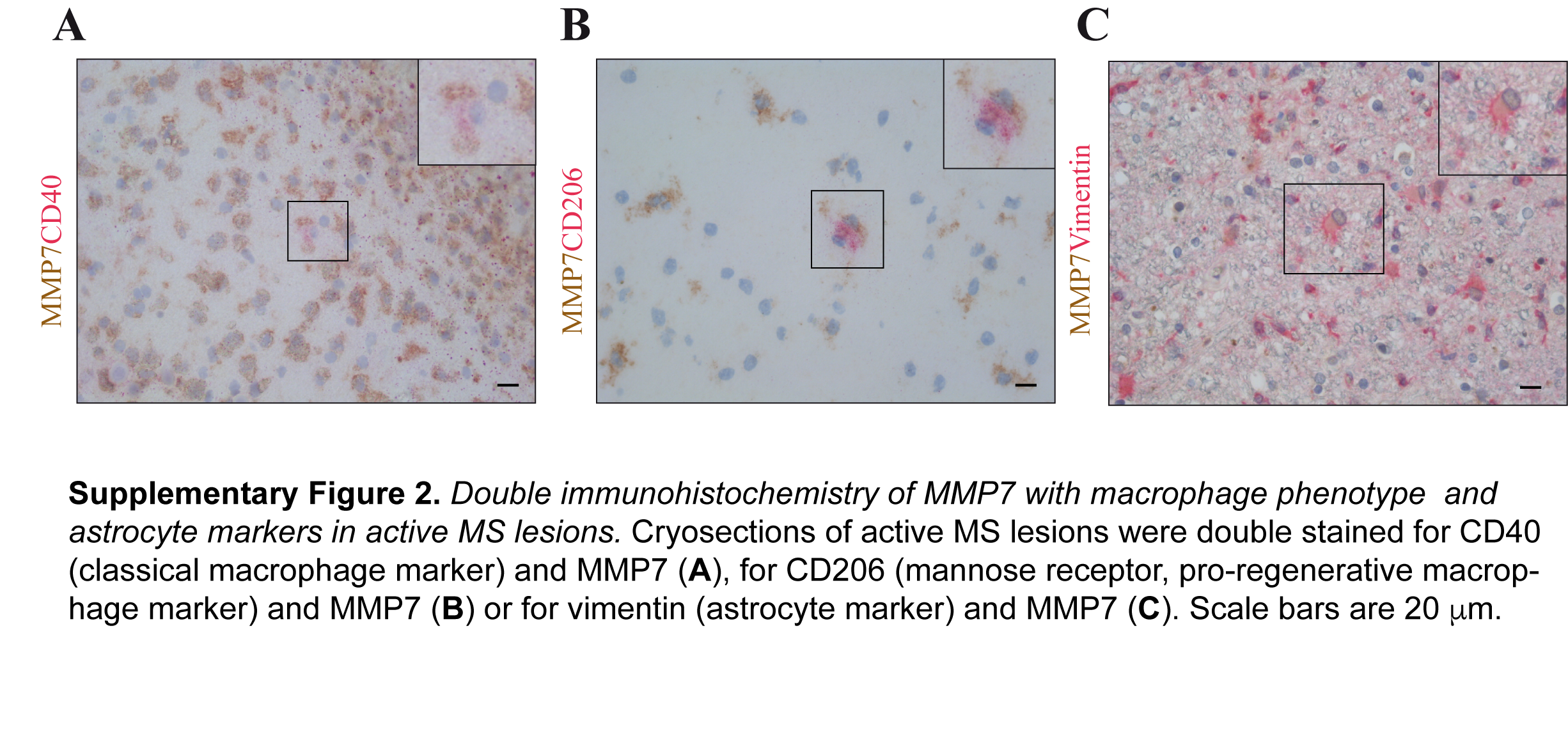

Supplement: Supplementary file 2 — Supplementary Figure 2 [file GLIA-66-1625-s002.tif]

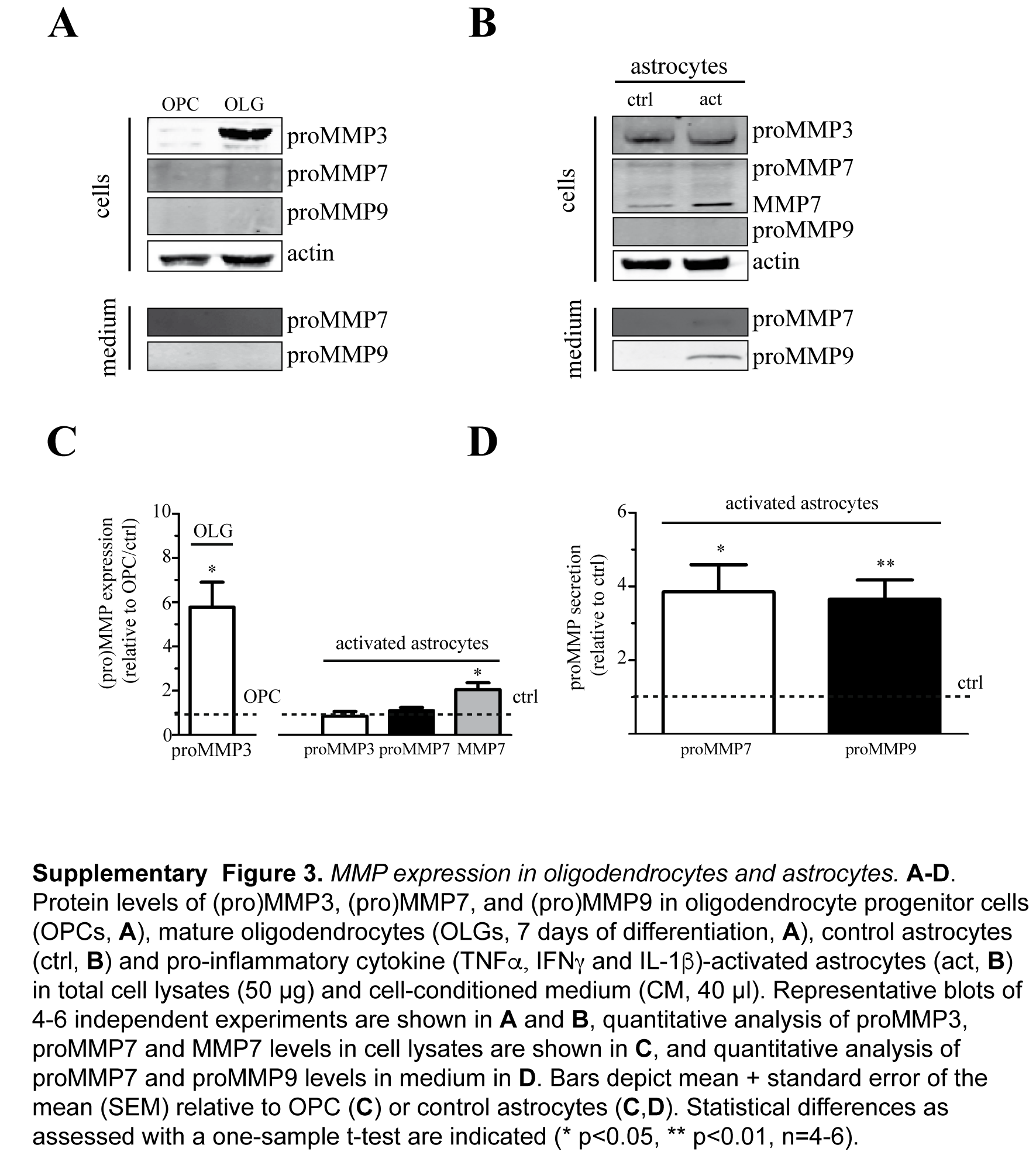

Supplement: Supplementary file 3 — Supplementary Figure 3 [file GLIA-66-1625-s003.tif]

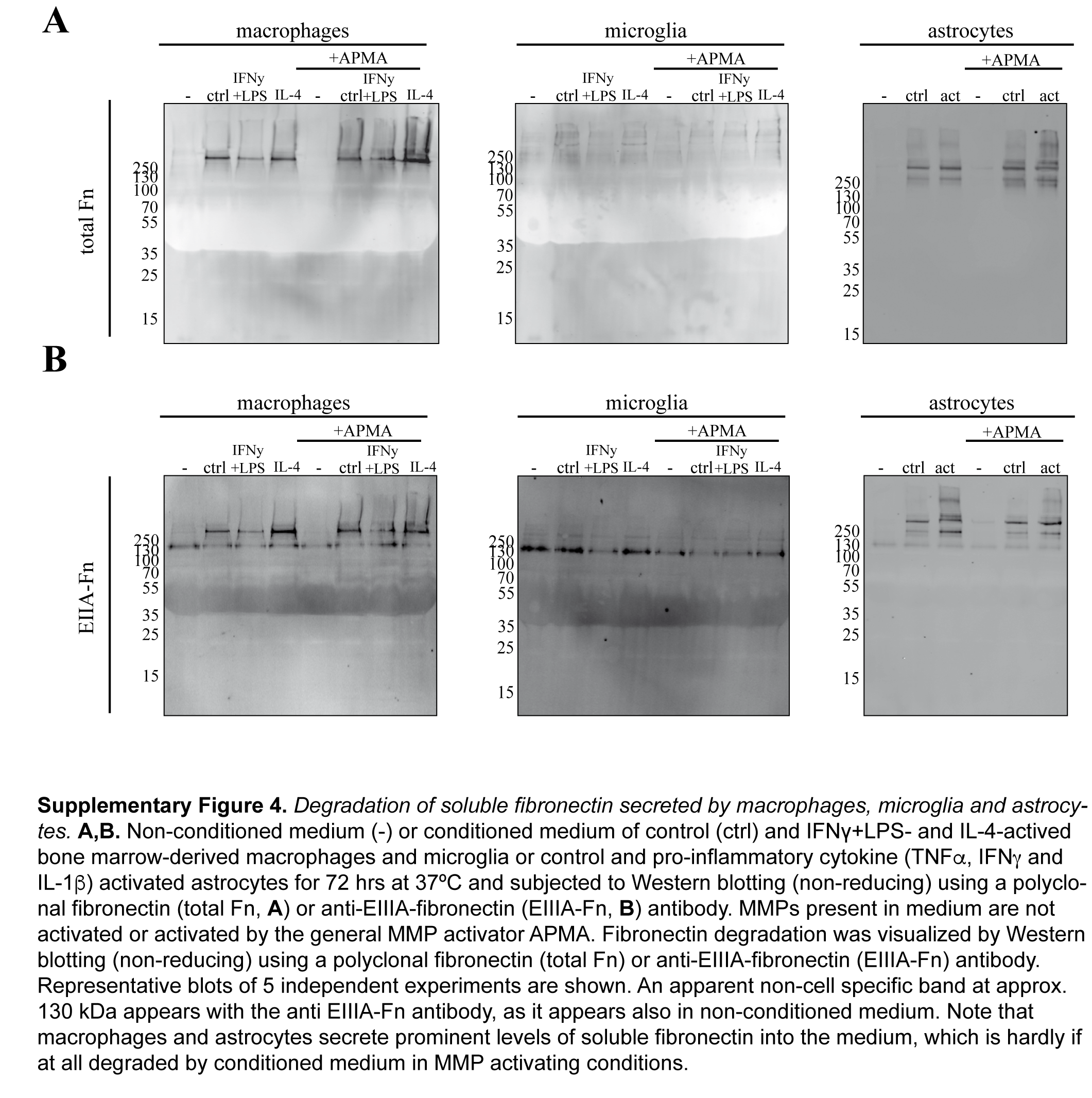

Supplement: Supplementary file 4 — Supplementary Figure 4 [file GLIA-66-1625-s004.tif]
